# Southern Dental Association

**Published:** 1888-01

**Authors:** 


### SOUTHERN DENTAL ASSOCIATION.

### NINETEENTH ANNUAL SESSION AT OLD POINT COMFORT, VA., 1887.

Reported for the Independent Practitioner by "Mrs. M. W. J."

Concluded From Page 650, Vol. VIII.

### THURSDAY EVENING SESSION—CONTINUED.

Prof. J. B. Hodgkins, Chairman of the Committee on Memorial, presented a report and resolution in memory of the late Dr. J. R. Walker, of New Orleans. After briefly sketching his honorable professional life, the report closed with the following :

*Resolved,* That this Association expresses its deep regret at the death of its late fellow-worker, Dr. J. R. Walker ; its profound sympathy for his widow and children, and a determination that the fruits of the seed he planted with us shall have a rich harvest in the prosperity of this Association.

*Resolved*, That a page of the minute-book be inscribed to his memory, and that a copy of these resolutions be sent to his widow and children.

The resolutions were unanimously adopted.

Resolutions of thanks were tendered the Virginia State Dental Society for their generous hospitality; to the S. S. White Company for facilities for clinics; to the Executive Committee for the admirable arrangements made for the meeting; to the proprietor of the Hygiea Hotel; to the railroads for reduced rates, etc.

The venerable Dr. Leslie, the originator of cohesive gold, was introduced by the President, and addressed the Association on that subject. He spoke of the claims made by others, and described the first contour filling ever made with cohesive gold. This gold was discovered by his brother, A. M. Leslie, and himself in 1838. In 1839, he spent a day in Louisville, Ky., where he made the first contour filling, in the mouth of Dr. N. Clouet. The correspondence which subsequently passed between them on the subject is still in existence as evidence of the truth of the claim made by Dr. Leslie.

The subject of Dental Hygiene was further discussed and finally passed.

Dr. McKellops, Chairman of the Committee on Voluntary Essays, read by title a paper which had been prepared for the meeting by Dr. J. R. Walker, during his last illness, upon "The Future of Dentistry—A Prophecy," and on motion it was ordered published in the proceedings.

Dr. W. H. Richards, of Knoxville, Tenn., thought that something should be done by the Association to direct the attention of the General Government to the care of the teeth of the common soldiers, and the appointment of dentists to the Army and Navy. The subject was not discussed.

Pathology and Therapeutics was next called, and Dr. W. C. Wardlaw, of Augusta, Ga., read a paper entitled "Neuralgia, its Association with Dental Lesions."

Dr. Wardlaw said that the term Neuralgia had been bandied about in the most indiscriminate manner, and was often used to conceal ignorance in reference to many aches and pains, the real nature of which was not understood, neuralgia being an entirely satisfactory explanation to many minds. As every painful effect must have an exciting cause, the object of Dr. Wardlaw's paper was to show that in a large majority of cases of neuralgic pains about the face and head, the specific origin was in dental lesions, and mostly from reflex irritation.

Pain is the only diagnostic symptom; the attacks are irregular, and the exciting cause often the most trivial. The dental lesions causing facial neuralgia are often most obscure in locality and character. Dr. Wardlaw briefly reviewed the anatomical structure and physiological relations of the parts involved, and the principles of reflex nervous action, demonstrating the possibility of neuralgia from dental irritation, and gave a long list of the lesions to which it is most frequently due, and the result of his observations in locating the lesion, and the corresponding point of attack. He finds that the lower molars, especially the third, give earache; the superior molars, headache; lesion of the bicuspids gives pain in the cheek and infra-orbital region ; neuralgic pains in the eye frequently accompany diseased cuspids or first bicuspids. He has known neuralgia of the posterior scalp and shoulder to proceed from a dead lateral incisor, etc. The radical treatment consists in the removal of the cause. In merely palliative treatment anodynes, liniments, etc., are useful. Systemic treatment may be required, with quinine in the periodical type. Bi-section of the tri-facial nerve, he says, is never necessary in pure facial neuralgia from dental irritation.

Dr. J. J. R. Patrick, of Belleville, Ills., explained his system of correcting irregularities, illustrated by a model and numerous diagrams. He explained the causes of many forms of irregularity of the teeth, cleft-palate, hare-lip, etc., in the unequal development of the bones of the face—excessive growth on the one hand, or the arrest of development on the other, perfect regularity resulting from the equal pace of growth of all the bones, the proper development of the alveolar process, and the eruption of the teeth at the proper point in the arch. He claimed that the deciduous teeth are thrown off like epithelial scales, like the hair of a horse, the shell of a crab, or the claws of lobsters. In living tissue, for absorption to take place as a physiological process, there must be an organ of absorption. If pus is absorbed we have septicemia, because there is no organ to finally dispose of it. As the glands of the mucous membrane throw off mucus internally, and the external skin throws off epithelial scales, so exuviation of the deciduous teeth takes place in a physiological manner. They are hardly formed before they begin to exuviate, each one bringing up its own process, the deciduous and permanent teeth being entirely independent of each other. The deciduous teeth are largest posteriorly, but the permanent teeth are twice their size laterally. It was a pleasing thought, but only a beautiful myth, that the deciduous teeth serve as guides for the permanent ones. The belief in an organ for the absorption of the roots of the deciduous teeth is dying out, for the question inevitably arises, what becomes of that organ when its work is done? Is there another behind to absorb it, and so on indefinitely? No, it is a process of exuviation. As the acid behind the horn of the stag causes it to fall off, so there is an acid in the mucus from the follicles in an abnormal condition which produces exuviation.

Dr. Patrick said that he was prepared to substantiate the assertion that the deciduous teeth have no more to do with the development of the permanent teeth than have the toe-nails; the proofs are as thick as blackberries. Anthropologists class the negro and the Australian as prognathous people, but there never was a prognathous negro child. While they have the deciduous teeth they have round, chubby faces. The change takes place with the eruption of the second growth, which differs entirely in form and shape, the bicuspids especially differing from the deciduous molars.

*Dr. W. H. Atkinson*—Said that in the removal of the deciduous teeth there was a return to embryonic conditions at the point of absorption, the *debris* being dissolved in the water of the tissues and taken up at the point of molecular metamorphosis by the lymphatics. He said there were, in the remarks just made, many fanciful generalizations, to which there was not time to reply.

*Dr. Patrick*—Said absorption was an indefinite word, which did not signify anything in particular; there were two kinds of absorption, external and internal; one of contruction and one of destruction; while the atmosphere is absorbing our tissues, we are absorbing that which lower organisms have built up for us. Cuvier has given us the term *exuviation* as a scientific term for a physiological process, which is not absorption, but is opposed to the pathological process, exfoliation.

*Dr. Atkinson*—Said this was making a discrimination without distinction; there was no such thing as external absorption. Exuviation was throwing off a corneous layer of epithelium, made possible by a process of desiccation; a throwing off of the fluid portions of the external walls of the cells. The lime-salts are melted down and floated to the mouths of the lymphatics and carried off.

At the close of this discussion, the election of officers, etc., took place (see page 561, October number), after which the Association adjourned till 8 P. M., when the paper of Dr. Wardlaw was taken up for discussion.

*Dr. Genese,* of Baltimore—Reported a case of neuralgia, due to dental lesion, in which he used, with the happiest effect, the solid extract of *Papaver Alba* as a sedative, securing to the patient the first night's rest enjoyed in several months. His sufferings had been extreme, and five sound teeth had been sacrificed in vain. The remaining teeth were of good structure, but close examination revealed softened dentine around an approximal cavity between the bicuspids, over a capped pulp, the unsuspected death of which had been the cause of the long suffering. From the time this was remedied there was no recurrence of the "neuralgia."

*Dr. R. Finly Hunt,* of Washington—While agreeing in the main with the paper, took exception to the use of the term *pure neuralgia* as applied to the result of dental lesions, pure neuralgia existing independently of any special local lesion, as an abnormal systemic condition. Here, as in so many other cases, we find the necessity for a more definite nomenclature.

Prof. L. G. Noel, of Nashville, next read a paper entitled "Etiology of Caries of the Teeth, Viewed from the Standpoint of Physiological Chemistry."

Dr. J. B. Hodgkin, of Baltimore, read a paper entitled "Are We Justified in Promising Success in Replantation?"

He doubted the probability that an extracted dry tooth, with lacunæ filled with desiccated and possibly decomposed protoplasmic mattter, can live again.

Dr. W. H. Morgan, of Nashville, and Dr. Geo. Winkler, of Augusta, described an experiment made with aqua ammonia upon cohesive gold, the former having maintained, while the latter doubted, that the cohesiveness would be destroyed. The experiment, though carefully made, was not thoroughly satisfactory.

*Dr. Morrison*, of St. Louis—In reply to Prof. Hodgkins' paper, said that he considered replantation in every way a legitimate operation. His successes so far outnumbered his failures that he felt justified in urging perseverance in the practice.

Dr. Stubblefield, of Nashville, read a paper on the "Histology of Hard Structures."

In this paper the writer disclaimed all pretensions to original research, attempting only to render more tangible the somewhat obscure, lengthy productions of the best authors concerning a field which, to the average dentist, is a *terra incognita*. That we know but little about the intimate primary structure and relations of the development of tissues, while the little that is regarded as certain is not clearly comprehended or fully appreciated by the average dentist, is largely due to lack of careful mental training and of thorough professional education. Histologists, as a rule, also, are not good teachers, and consequently fail to present clearly and concisely what they ascertain, losing themselves in a maze of wordy windings through thickets of technicalities and interminable circumlocutions.

The paper, though as brief and concise as possible, in an attempt to cover the whole field of the histology of hard tooth structure, beginning with the formation of the nucleus of the bone-cell, consumed the time till nearly midnight. The Association then adjourned to meet on board the steamer *en route* to Washington City, a recess being taken to enable the members to participate in the deliberations of the Dental Section of the International Medical Congress, before final adjournment.

Saturday, Sept. 3d, the Association was called to order on board the steamer, where the ceremonies of the installation of the officers for the coming year took place in due form, accompanied by rather more than the usual speech-making, and by other pleasant social features, due to the unusual surroundings. Reaching Washington soon after dark, carriages, omnibuses and street cars were in waiting to convey each and all to the lodgings secured in advance by the untiring efforts of Dr. R. Finley Hunt.

At the close of the Congress, the Southern Dental Association adjourned to meet again in Louisville, Ky., in joint session with the American Dental Association, in August, 1888.

